# Validity of self‐reporting depression in the Tabari cohort study population

**DOI:** 10.1002/npr2.12138

**Published:** 2020-09-19

**Authors:** Mehran Zarghami, Fatemeh Taghizadeh, Mahmood Moosazadeh, Motahhareh Kheradmand, Keyvan Heydari

**Affiliations:** ^1^ Department of Psychiatry School of Medicine, Psychiatry and Behavioral Sciences Research Center Addiction Institute Mazandaran University of Medical Sciences Sari Iran; ^2^ Student Research Committee, Faculty of Medicine, Psychiatry and Behavioral Science Research Center Addiction Institute Mazandaran University of Medical Sciences Sari Iran; ^3^ Health Sciences Research Center Addiction Institute Mazandaran University of Medical Sciences Sari Iran; ^4^ Student Research Committee, School of Medicine Gastrointestinal Cancer Research Center, Non‐Communicable Diseases Institute Mazandaran University of Medical Sciences Sari Iran

**Keywords:** beck depression inventory, depression, self‐reporting

## Abstract

**Aims:**

Depression is a common cause of mortality and morbidity worldwide. To detect depression, we compared BDI‐II scoring as a valid tool with participants' self‐reporting depression.

**Methods:**

The sample size was determined to include 155 participants with positive self‐reporting of depression in a total of 1300 samples with 310 healthy participants were included in the study through random selection. In order to evaluate the diagnostic value of self‐reporting, BDI‐II was completed by blind interviewing to the case group as well as to another group who reported that they were not depressed, as control.

**Results:**

Sensitivity, specificity, accuracy, false positive, false negative, positive, and negative predictive values of self‐reporting were calculated 58.4%, 79.1%,73.4%, 20.8%, 41.6%, 51.8%, and 83.2% for the total population, respectively, as well as, sensitivity, specificity, accuracy, positive, and negative predictive values of self‐report in males were 83.3%, 77.2%, 77.1%, 43.8%, and 95.6% and 53.7%, 78.1%, 71.2%, 49.2%, and 81.1% for females, respectively.

**Conclusion:**

The positive predictive value and sensitivity of self‐reporting are insufficient in total population and females, and therefore self‐reporting cannot detect depressed patients, but regarding to its average positive predictive value, perhaps, it can be used to identify nondepressant individuals.

## Abstract 2

The positive predictive value and sensitivity of self‐reporting are insufficient in total population and females, and therefore self‐reporting cannot detect depressed patients, but regarding its average positive predictive value, perhaps, it can be used to identify nondepressant individuals.

## Graphical abstract

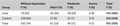

## 1 INTRODUCTION

The World Health Organization (WHO) has identified depression as the fourth reason of disability in the world, accounting for the greater portion of nonlethal diseases, and predicts it to be the second cause of death by 2020^1^, ^2^, ^3^. In a review study, the prevalence of lifetime depression varied from 1.5 percent in Taiwan to 19 percent in Lebanon. The average in western Germany was 9.2 percent, and in Edmonton in Canada, it was reported at 9.6 percent^1^. An international research by the WHO, reported the prevalence of major depression in the general population to be from 1 percent in the Czech Republic to 16.9 percent in the United States, with an average of 8.3 percent in Canada, and up to 9 percent in Chile^1^. The average global prevalence of depression is reported to be about 15 percent^4^. The prevalence of depression in the Iranian adult population is assessed at 21 percent^4^. Regarding the high importance of this disorder, screening of this serious condition and timely management would be an important subject. There are several assessments for diagnosis of depression, namely Hamilton Depression Rating Scale (HAM‐D), Zung Self‐Rating Depression Scale^5^, Montgomery‐Asberg Depression Rating Scale, HADS^6^, Geriatric Depression Scale, and the General Health Questionnaire (GHQ). They have few items for depression, except the HAM‐D^4^, these depression assessment tools were developed as a measure of treatment outcome rather than a diagnostic or screening depression^1^. However, the Beck Depression Inventory (BDI) assesses both the psychosomatic and the physical symptoms, and its effectiveness has been discussed in many studies^7^. This tool has been used in more than 7000 researches so far. The theoretical assumption of the BDI relied upon the negative believes that distorted cognition is the core of depression characteristic^2^. This inventory is a valuable instrument, with high reliability to discriminate depressed and nondepressed participants, and its content, structural, and concurrent validity have been approved^2^. This tool has been revised two times, and the latest version (BDI‐II) was published in 1996^3^. The available psychometric evidence showed that the BDI‐II could be noticed as a valid cost‐effective inventory for measuring the depression severity, with wide applicability for research and clinical practice^2^.

We administrated BDI‐II as screening tool for assessment of depression after the self‐reporting of depression in the Persian cohort in Mazandaran, Iran. As in some studies, it has been indicated that the prevalence of depression measured through diagnostic scales by patients has been higher than the self‐report results^2^, the researchers decided to compare the diagnostic value of the depression with BDI‐II in Mazandaran's Persian cohort study with self‐reported depression. The study's primary objectives were sensitivity, specificity, accuracy, false positive, false negative, positive, and negative predictive values of self‐reporting, and the secondary outcomes were the association of depression according to BDI‐II with sex, age, depression, depression in family in the case and control groups.

To our knowledge, this is the first study to compare the prevalence of depression with self‐reporting and BDI‐II as well as the first study to evaluate depression screening in a general population with the patients' self‐report.

## 2 METHODS

In this cross‐sectional study, we used a subset of data collected in Tabari cohort (Mazandaran's Persian cohort study), which is part of the national cohort, entitled as Prospective Epidemiological Research in Iran (Persian)^4^, ^5^.

For conducting this study, 1300 participants with self‐reporting depression interview, aged 35‐70 years living in urban areas of Sari, Mazandaran, Iran, were enrolled. As part of data collection in Tabari cohort, a standardized questionnaire consisting of general information, socioeconomic status, and occupational history was completed. All the participants were asked a question" Are you depressed?”. Among all the participants, 155 cases had a positive history of depression, which were selected as the case group. Among the remaining participants who did not report depression, 310 individuals were selected as control group randomly and matched in age and sex.

In order to evaluate the diagnostic value of self‐reporting, BDI‐II was completed to the case group as well as to another group who reported that they were not depressed.

Trained interviewers who were blind to the interviewees, dispatched to the households based on their Household Registry Number addresses to fill out the demographic questionnaire and the BDI‐II. For illiterate participants, the questions were read and they answered without any elaborations or comments.

This cross‐sectional study aimed to investigate the diagnostic values of self‐reporting in patients with depression compared to BDI‐II in Mazandaran's Persian cohort study with a total of 1300 samples. The sample size was determined to include 116 participants based on the results of Kim et al study^3^, where the correct classification is reported to be 82 percent, with a confidence level of 95% and an accuracy of 0.07. With the effect size equal to 1.3 times, the sample size was estimated 155 participants that allocated through the census method. In order to increase the test power by 2 times, 310 healthy participants (by self‐reporting) were entered the study through random selection (based on the available list) and the following formula:

$$N=\frac{Z^{2}*P\left(1‐P\right)}{d^{2}}$$

### 2.1 Statistical analysis

Data were entered into SPSS (version 22) software for statistical analysis. After filtering, the distribution of characteristics of the studied population was presented through descriptive tests such as frequency, mean, and standard deviation. Comparison between three groups for categorical data were statistically analyzed using chi‐ square or Fisher‐exact test. Also, sensitivity, specificity, positive, and negative predictive value and accuracy of self‐report method were determined. A p‐value of 0.05 or less was considered significant statistically. Using IBM SPSS12 statistics version 23 and Stata version, the data were analyzed.

### 2.2 Questionnaires

#### 2.2.1 Demographic information Questionnaire

This questionnaire included demographic information such as age, sex, and history of depression.

#### 2.2.2 The Beck Depression Inventory (BDI‐II)

The BDI‐II is a multiple‐choice self‐report inventory, consisting of 21 questions, first developed by Aaron Beck in 1961^6^. The 21 items are based on symptoms follow as:

1. Sadness 2. Pessimism 3. Sense of failure 4. Lack of satisfaction 5. Guilt 6. Feelings of being punished 7. Self‐hate, 8. Self‐accusations, 9. Self‐harm, 10. Crying spells (crying periods), 11. Early suffering (excitability), 12. Social isolation, 13. Undecidedness, 14. Self‐thought (change in body image), 15. Weakness and slowness (slowness in doing a task, slowness at work), 16. Sleep disturbance (insomnia), 17. Fatigue 18, decreased appetite (loss of appetite), 19. Weight loss, 20. Somatic preoccupation, and 21. Loss of libido^8^, ^9^.

In this inventory, 4‐6 questions are asked concerning each of the mentioned items based on one of the symptoms of the illness, ranging from the mildest to the most severe aspect of the mentioned attribute^9^. The quantitative values of each item from 0 to 3 are determined as mild to severe disorder. Several forms of this questionnaire have been prepared. Here, the regular form includes 21 items^9^.This questionnaire is a self‐ assessment instrument and takes 5‐10 minutes to complete.

### 2.3 Scoring

The total score ranges from 0 to 63. These marks are interpreted in the diagnosis of depression as follows: normal (no clinical disease (1‐13), mild depression (14‐19), moderate depression (20‐28), and severe depression (29‐63)^9^.

It should be noted that, even though this inventory was designed for use in clinical populations^10^; besides, it could also be used in normal populations^9^,^11^.

### 2.4 Reliability and validity

Beck, Stier, and Garbin obtained the internal consistency coefficients at 0.73‐0.92, with an average of 0.86^14^. The content of the BDI materials included six of the nine categories of DSM‐III for diagnosis of depression^2^. The correlation of this test with the Hamilton scale for depression (0.73), Zong's depression scale (0.76), and MMPI depression scale (0.76) was obtained^14^. The correlation coefficient was obtained as 0.54 through the MMPI Depression Scale^12^, ^13^. However, factor analysis showed a robust dimension of general depression composed by two constructs: cognitive‐affective and somatic‐vegetative^2^.These data support the reliability and concurrent validity of the BDI‐II‐Persian as a measure of depressive symptoms in nonclinical samples^8^.

### 2.5 Cut‐off of BDI‐II

The cut‐off score for screening of depression varied according to the type of sample. In a study in Iran, the best BDI‐II cut‐off was 14, with sensitivity of 62% (95% CI (43%, 81%)), specificity of 81% (95% CI (72%, 90%)), PPV of 53%, and NPV of 85%^8^. The internal consistency was described as around 0.9 and the test‐retest reliability ranging from 0.73 to 0.96^2^. Accordingly, in this study, a score of 14 was considered as the cut‐off point for screening of depression.

## 3 RESULTS

### 3.1 Biographic characteristics of population

141 (32%) of participates were cases, and 310 (68%) of them were controls (Table 1). Of all the participants, 69 (15%) were male and 382 (85%) female, 437(96%) married, 10 (2%) widowed, 3 single, and 1 divorced. With regard to age, 136 (30%) of the participants were 37‐46 years old, 178 (39%) 47‐56 years old, 117 (25%) 57‐66 years old, and 20 (4%) 66‐72 years old.

**Table 1:** Frequency of population characteristics (sex, age, being under depression treatment, existence of depression in first‐grade relatives) in the case and control groups based on self‐report

Group	Case *F* (%)	Control *F* (%)	Total *F* (%)
Male	23 (16)	46 (15)	69 (15)
Female	118 (84)	264 (85)	382 (85)
Total	141 (100)	310 (100)	451 (100)
Age group	Case *F* (%)	Control *F* (%)	Total *F* (%)
35‐50	57 (41)	125 (41)	182 (40)
51‐72	84 (59)	185 (59)	269 (59)
Total	141 (100)	310 (100)	451 (100)

### 3.2 Sensitivity, specificity, positive, and negative predictive values

With the cut‐off 14 of BDI‐II, sensitivity, specificity, accuracy, false positive, false negative, positive, and negative predictive values of self‐reporting were calculated 58.4%, 79.1%,73.4%, 20.8%, 41.6%, 51.8%, and 83.2% for the total population, respectively.

Sensitivity, specificity, accuracy, positive, and negative predictive values of self‐report in males were 83.3%, 77.2%, 77.1%, 43.8%, and 95.6% and 53.7%, 78.1%, 71.2%, 49.2%, and 81.1% for females, respectively.

In addition, sensitivity, specificity, accuracy, positive, and negative predictive values of self‐report was 79.2%, 75.9%, 76.4%, 33.3%, and 96% for the 35‐50 age group, and 51%, 79.8%, 69.5%, 58.3%, and 74.6% for the 51‐72 age group, respectively.

In addition, Table 1 shows the frequency of population characteristics in the case and control groups based on self‐report; moreover, Table 2 shows the frequency of depression according to BDI‐II (sex, age, depression, depression in family) in the case and control groups according to BDI‐II, respectively. Table 3 presents the frequency of depression in the case and control groups based on BDI‐II.

**Table 2:** Distribution of sex, age, family history of depression, being depressed according to BDI‐II, and being under treatment of depression

Gender	Without depression *F* (%)	Depressed *F* (%)	Total *F* (%)
Male	57 (18)	12 (10)	69 (15)
Female	274 (82)	108 (90)	382 (84)
Total *F* (%)	331 (100)	120 (100)	451 (100)
Age group	Without depression *F* (%)	Depressed *F* (%)	Total *F* (%)
35‐50	158 (47)	24 (20)	182 (40)
51‐72	173 (52)	96 (80)	269 (59)
Total *F* (%)	331 (100)	120 (100)	451 (100)
Being under depression treatment	Without depression *F* (%)	Depressed *F* (%)	Total *F* (%)
No	273 (82)	57 (47)	330 (73)
Yes	58 (17)	63 (52)	121 (26)
Total *F* (%)	331 (100)	120 (100)	451 (100)
Family history of depression	Without depression *F* (%)	Depressed *F* (%)	Total *F* (%)
No	298 (90)	107 (89)	405 (89)
Yes	33 (10)	13 (10)	46 (10)
Total *F* (%)	330 (100)	120 (100)	451 (100)

**Table 3:** Frequency of depression in the case and control groups based on BDI‐II

Group	Without depression, *F* (%)	Mild, *F* (%)	Moderate, *F* (%)	Severe, *F* (%)	Total, *F* (%)	*P* value
Case	73 (52)	34 (24)	28 (20)	6 (4)	141 (100)	.001
Control	258 (84)	27 (8)	22 (7)	3 (1)	310 (100)	
Total	331 (74)	61 (13)	50 (11)	9 (1)	451 (100)	

## 4 DISCUSSION

In this study, the prevalence of depression was assessed blindly (being case or control) using BDI‐II in two groups. According to the results, the sensitivity and specificity of self‐reporting were found to be low, with many of the cases being found not depressed via BDI‐II (Table 3). It was concluded that self‐reporting was not suitable for screening for depression in this population, and thus, there is a need to use a scale such as BDI‐II as the gold standard^9^ for depression screening.

Individual clinical interview is the "gold standard" for diagnosis of depression^14^. However, this approach may be problematic for screening of depression in large populations. In the Persian cohort study, the participants were asked only one question in this case, namely “are you depressed based on physician's opinion?”. This study aimed to evaluate the diagnostic value of self‐reporting compared with one of the most popular scales for depression screening.

The BDI is one of the most well‐known tools for screening of depression in general population and psychiatric patients^14^, ^15^. One of the problems of the BDI is that it did not completely include all of the symptoms in the DSM in depression criteria^16^. This revised instrument does not rely on any certain theory of depression^14^. The **BDI**‐II has a good reliability and validity^10^. The correlation between BDI‐II and BDI‐I has been described strong^17^.

The correlation between BDI‐II and BDI‐I has been reported high^2^ With respect to the Multiscale Depression Inventory as a “gold standard”^18^, the curve of receiver operational characteristic showed **BDI**‐II to be an adequate diagnostic measure^19^ and that the optimal total cut‐off score was 18.5^17^ With this cut‐off score, 25% of multiple sclerosis patients were positively identified as having clinically relevant depression. The result of this study showed that the **BDI**‐II is a valid, reliable, and simple tool for depression detecting and grading^17^. In a systematic review study on psychometric BDI‐II characteristics, 118 studies were assigned into three groups: nonclinical, medical participants, psychiatric, or institutionalized participants. The internal consistency was obtained 0.9, and the test‐retest reliability was revealed 0.73‐0.96. The cut‐off score for depression screening varied according to the variety of participants. Factor analysis presented a strong dimension of general depression composition with 2 constructs: somatic‐vegetative and cognitive‐affective^2^. The **BDI**‐II is a valid psychometric instrument^18^, showing high reliability, capacity to discriminate between depressed and nondepressed subjects^20^, ^21^, ^22^, ^28^, and improved concurrent, content, and structural validity. Based on the available psychometric evidence, the **BDI**‐II can be viewed as a cost‐effective instrument for measuring the severity of **depression**, with broad applicability for research and clinical practice worldwide^2^. **BDI‐**II instrument depends on the clinical and social context of the assessment^24^. This questionnaire was used in our study for depression screening in the general population.

Concerning the self‐report in the research, in a study, self‐reported alcohol use was compared to biomarker tests via the Audit and 90‐day recall for 193 women from prenatal clinics. The Audit was positive in 67.9% of the participants, and 65.3% of them directly reported drinking. Individual biomarkers revealed less drinking than self‐reporting, but 64.8% had drinking‐positive values on biomarkers, which were not different significantly from self‐report. The biomarkers showed that 3.1%‐6.8% of participants lied about their drinking. The combined biomarker sensitivity was 95%‐80% and the specificity was 49%−76% for drinking in the 7‐90 days ago. The best yield combined biomarker results were 89.6% with accuracy of 78.8% when evaluating 90 days drinking^25^. In confirmation of the conclusion of this study, many of the patients may have given contradictory answers or lied in self‐report regarding their depression, or otherwise gave vague or ambiguous answers.

Another study evaluated the interactions among three selected FKBP5 single nucleotide polymorphisms and objectively recorded ELS and self‐reported early life stress (ELS) related to depression symptoms in midlife. The participants completed the Beck Depression Inventory at ages of 61.5 years (time 1) and 63.4 years (time 2); 165 and 181 participants were separated from their parents in childhood as a result of evacuations during World War II as indicated by self‐reports and the Finnish National Archives registry, respectively. The relationship between objectively recorded ELS and self‐reporting, and the average BDI score (mean of time 1 and time 2) or mild to severe **BDI** scores, or both, were moderated by the FKBP5 variants.FKBP5 variations combined with and objectively recorded ELS and self‐reporting could predict more noticeable depression symptoms in midlife^26^.

Moreover, in South Africa, 5059 participants aged above 40 years were entered in a study from 2014 to 2015. HIV biomarker testing, self‐reporting HIV status, and dried bloodspots were found during interviews at home. Regarding the biomarker results, 50.9% of participants reported knowing their HIV status and reported that accurately. PPV of self‐reporting was 94.1%, NPV was 87.2%, specificity of 99%, and sensitivity of 51.2%. The patients on ART were more likely to reporting their HIV positive status, and the patients that reporting false negatives were more likely to have older HIV tests. False‐negative reports were mostly explained by lack of the testing, suggesting to be retreating HIV stigma in this setting^27^. It seems that drinking alcohol, cigarette smoking^28^ and HIV infection may be reflected as a stigma which can predict high rate of negative self‐report. Concerning the results of this study, the stigma of having psychiatric disorders^29^, such as depression, is a barrier to self‐reporting of these problems, and a valid and reliable instrument is required to be arranged and conducted for detecting depression. In addition, sensitivity in our study was low by self‐reporting compared with BDI‐II as a gold standard.

The positive predictive value and sensitivity of self‐reporting are low, and therefore self‐reporting cannot help in detecting depressed patients; however, concerning its average positive predictive value, perhaps, it can be used to identify nondepressant people.

## 5 LIMITATIONS

The present study does have some limitations. First, it is difficult to launch a causal association in a cross‐sectional study. Second, depression was measured by the BDI‐II and self‐reporting rather than a psychiatric structured interview. The BDI is a self‐report questionnaire, which might underestimate a person's grade of depression. Moreover, many persons were excluded because they lacked a self‐reporting depression. There was a possibility that those persons who were affected by mild or moderate depression did not identify as depressed person.

## CONFLICT OF INTEREST

The authors declare no conflict of interest.

## AUTHOR CONTRIBUTIONS

MZ developed the original idea for the trial and attracted funding. FT, MM, MZ, and MKH were responsible for the design of the study protocol. MM performed the statistical analyses. FT, MM, KH, MZ, and MKH conducted interpretations. FT developed the first draft of the paper. MM, MZ, and MKH revised for important intellectual content. All authors read and approved the final version.

## APPROVAL OF THE RESEARCH PROTOCOL BY AN INSTITUTIONAL REVIEWER BOARD

The protocol for this research project has been approved by a suitably constituted Ethics Committee of the institution and it conforms to the provisions of the Declaration of Helsinki. Committee of Mazandaran University of Medical sciences, Approval No. IR.MAZUMS.REC.95.2707.

## INFORMED CONSENT

The clients were explained on the purpose and method of the study. They were asked to complete a consent form.

## REGISTRY AND THE REGISTRATION NO. OF THE STUDY

N/A

## ANIMAL STUDIES

N/A

## Data Availability

The data that support the findings of this study are available on request from the corresponding author. The data are not publicly available due to privacy or ethical restrictions. Because we did not obtain informed consent on the public availability of raw data.
